# Diabetes Mellitus Predicts Weight Gain After Surgery in Patients With Acromegaly

**DOI:** 10.3389/fendo.2022.854931

**Published:** 2022-03-09

**Authors:** Han Na Jang, Yong Hwy Kim, Jung Hee Kim

**Affiliations:** ^1^Department of Internal Medicine, Seoul National University College of Medicine, Seoul National University Hospital, Seoul, South Korea; ^2^Department of Neurosurgery, Seoul National University College of Medicine, Seoul National University Hospital, Seoul, South Korea; ^3^Pituitary Center, Seoul National University Hospital, Seoul, South Korea

**Keywords:** acromegaly, growth hormone, fat metabolism, insulin resistance, adipokine

## Abstract

**Objective:**

Metabolic complications are common in patients with acromegaly. However, this occasionally does not improve post-surgery and may be related to postoperative weight gain. We aimed to investigate the postoperative weight change and factors associated with postoperative weight gain in patients with acromegaly.

**Design and Methods:**

Overall, 113 consecutive patients with body weight records pre- and 3–6 months post-surgery between October 2009 and March 2021 were enrolled. Patients were divided into three groups: weight loss (weight decrease ≥3%), stable, and weight gain (weight increase ≥3%). Hormone status, metabolic comorbidities, and anthropometric parameters were compared between the groups.

**Results:**

Among 113 patients, 29 (25.7%) and 26 (23.0%) patients lost and gained weight, respectively, post-surgery. There were no significant differences in baseline characteristics, including age at diagnosis, sex, body mass index, and growth hormone levels among the three groups. The prevalence of diabetes mellitus at diagnosis was significantly higher in the weight gain group than in the other groups. Patients with diabetes (n=22) had a 5.2-fold higher risk of postoperative weight gain than those with normal glucose tolerance (n=37) (P=0.006). In the diabetes mellitus group, the percentage lean mass decreased (-4.5 [-6.6–2.0]%, P=0.002), and the percentage fat mass significantly increased post-surgery (18.0 [4.6–36.6]%, P=0.003), whereas the normal glucose tolerance group did not show body composition changes post-surgery.

**Conclusion:**

In patients with acromegaly, 23% experienced ≥3% weight gain post-surgery. Diabetes mellitus at diagnosis is a significant predictor of weight and fat gain post-surgery.

## Introduction

Acromegaly is caused by excessive growth hormone (GH) secretion, mainly by pituitary adenomas (1). Under the influence of GH and insulin-like growth factor-1 (IGF-1), patients with acromegaly show a change in appearance and have several systemic complications such as cardiovascular, respiratory, and metabolic complications (1–5). Although insulin resistance and diabetes mellitus (DM) are associated with increased adiposity, patients with acromegaly exhibit increased insulin resistance despite a decrease in fat mass and increased muscle mass. As a putative mechanism, GH has a lipolytic effect by inducing the hydrolysis of triglycerides in adipose tissues, thereby increasing the production of free fatty acids and contributing to insulin resistance (6). In addition, GH itself reduces peripheral glucose uptake in the skeletal muscle and increases hepatic glucose production through gluconeogenesis, inhibiting pancreatic β-cell function and insulin secretion (7–9).

Therefore, it was expected that complications related to acromegaly would improve when GH levels normalized after surgery. Several studies have reported improvements in hypertension, cardiomyopathy, and DM after treatment (10–12). However, hypertension, DM, and obstructive sleep apnea did not improve after treatment in some patients (13, 14). The lack of improvement in postoperative metabolic complications may be related to weight gain after surgery (14). In this regard, several studies have reported that body weight and fat mass increase after acromegaly treatment (15–19). On the other hand, some studies have shown stable weight after treatment in patients with acromegaly (10).

Herein, we aimed to investigate the change in weight and body composition after surgery and elucidate predictors of postoperative weight gain to improve long-term outcomes in patients with acromegaly.

## Materials and Methods

### Study Patients

We included consecutive patients diagnosed with acromegaly based on oral glucose tolerance test (OGTT) and underwent surgery between October 2009 and March 2021 at the Seoul National University Hospital. The medical records of patients who underwent surgery between October 2009 and December 2013 (n=67) were reviewed retrospectively. From 2014, we analyzed the data of patients enrolled in the Seoul National University Pituitary Disease Cohort Study (ClinicalTrials.gov, protocol ID 1503-040-654) according to the pre-established protocol (n=100). We excluded patients who lost followed up within 3 months after surgery. Of the 167 patients with postoperative follow-up data, we further excluded patients with a history of previous pituitary surgery, radiotherapy to pituitary gland, or administration of somatostatin analogs before surgery (n=5), and those without weight information after surgery (n=49). In the final analysis, a total of 113 patients were included. Clinical information was obtained before and 3–6 months after surgery.

Weight gain was defined as an increase of ≥3% compared to before surgery, weight loss was defined as a decrease of ≥3%, and the stable weight was defined as weight change falling between these parameters. DM was defined as having an HbA1c level of ≥6.5% or use of antidiabetic drugs; prediabetes was defined as an HbA1c level of 5.7–6.5%, and normal glucose tolerance (NGT) was defined as HbA1c level of <5.7%. In addition, other comorbidities, such as hypertension, dyslipidemia, cardiovascular disease, stroke, osteoporosis, sleep apnea, arthralgia, and carpal tunnel syndrome, were defined based on medical records.

### Anthropometric Measure/Body Composition Analysis

Anthropometric measurements were measured using bioelectrical impedance analysis (Inbody 970, Seoul, South Korea). Height (cm), body weight (kg), waist circumference (cm), hip circumference (cm), lean mass (kg), and fat mass (kg) were measured. In addition, body mass index (BMI) (kg/m2), waist-to-hip ratio, percentage lean mass (%), percentage fat mass (%), and visceral fat area (cm2) were calculated. The percentage lean mass (%) and percentage fat mass (%) are the ratios of lean mass and fat mass to body weight, respectively.

### Hormone Assessment/Other Clinical Parameters

Hormone assessment was performed before surgery and 3–6 months after surgery. An OGTT was performed by measuring glucose and GH before, 1 and 2 h after administering 75 g glucose. Nadir GH levels were the lowest measured GH during the OGTT; IGF-1 levels were expressed as absolute values or the fold-change of the upper limit of the age- and sex-specific normal range (ULN). The age-specific reference ranges of IGF-1 were: 21–30 years, 232–385 ng/mL; 31–40 years, 177–382 ng/mL; 41–50 years, 124–290 ng/mL; 51–60 years, 71–263 ng/mL; 61–70 years, 94–269 ng/mL, 71–80 years, 76–160 ng/mL. Acromegaly was diagnosed based on the non-suppressed GH response on the OGTT and a high IGF-1 level above the age- and sex-specific reference range (20). Remission was defined as GH levels suppressed to <1 ng/mL during the OGTT, and IGF-1 within the age- and sex-specific reference range (21).

TSH (thyroid-stimulating hormone) deficiency was defined as a low or normal TSH level (reference rage, 0.4–4.1 μIU/mL) with low free T4 (<0.70 ng/dL). Adrenocorticotropic hormone (ACTH) deficiency was defined as a peak cortisol level <18 μg/dL after a short Synacthen test, or when ACTH levels were low or in the normal range (reference range, 10-65 pg/mL) with a low morning cortisol level (≤5 μg/dL). Normal menopause was defined as follicle-stimulating hormone (FSH) levels >30 mIU/mL with estradiol (E2) <50 pg/mL. FSH/luteinizing hormone (LH) deficiency was defined as testosterone or E2 levels were lower than normal with low or normal FSH and LH levels. Furthermore, central diabetes insipidus (CDI) was defined as requiring desmopressin administration.

Fasting plasma glucose, insulin, and total cholesterol levels were measured after fasting for more than 8 hours. In addition, 1-hour and 2-hour-postprandial glucose levels were measured 1 and 2 hours after OGTT, respectively. A homeostatic model assessment for insulin resistance (HOMA-IR) was obtained by dividing the product of fasting insulin (mIU/L) and fasting blood glucose (mg/dL) by 405 (22). Systolic and diastolic blood pressures were defined as the average of three blood pressures measured after hospitalization for hospitalized patients.

Maximal tumor size was defined as the largest size among width, length and height on T1-weighted contrast-enhanced magnetic resonance imaging.

### Hormone Assay

GH was measured with an immunoradiometric assay kit (Izotop, Budapest, Hungry), and the intra- and inter-assay coefficient of variation (CVs) were 1.5–3.5% and 2.5–3.3%, respectively. IGF-1 was also measured with an immunoradiometric assay kit (Beckman Coulter, Brea, California, USA), the intra- and inter-assay CVs were <5.6% and <8.3%, respectively. The lowest detectable levels of GH and IGF-1 were 0.02 ng/mL and 4.55 ng/mL, respectively: we used the World Health Organization international standard measurement for GH (98/574) and IGF-1 (91/554). IGF-1 was expressed as a multiple of the upper limit of the normal. LH, FSH, E2, testosterone, prolactin, free T4, TSH, ACTH, and cortisol levels were measured between 8 and 10 am by radioimmunoassay and immunoradiometric assay.

### Surgical Procedures

Details of the surgical procedures were similar to those in our previous report (23). The operation was initiated through the binostrial pure endonasal or transseptal route without resection of the turbinates or posterior septectomy with an endoscope. Extracapsular dissection was the standard procedure, and sharp dissection with microdissectors was performed in case without a definite pseudocapsule.

### Statistical Analysis

Continuous variables with a normal distribution are expressed as mean and standard deviation, and variables with a non-normal distribution are expressed as median [interquartile range (IQR)]. Repeated-measures analysis of variance (ANOVA) and the Bonferroni multiple comparison test for *post hoc* analyses (the Friedman test and Dunn multiple comparison test for *post hoc* analyses were used to analyze non-normally distributed continuous variables) were used to compare patients according to weight change and glycemic status. Changes in clinical information before and after surgery were analyzed using paired t-test and Wilcoxon signed-rank test. The correlation between weight change and clinical information was analyzed using Pearson correlation analysis for parametric analysis and Spearman analysis for non-parametric analysis. The relationship between glycemic status and weight gain was analyzed using logistic regression analysis. Statistical significance was set at P <0.05. SPSS for Windows version 25.0 (IBM Co., Armonk, NY, USA) and Prism 5 for Windows version 5.03 (GraphPad Software, San Diego, CA, USA) were used for statistical analysis.

### Ethical Statement

This study was conducted in accordance with the principles of the Declaration of Helsinki. The trial protocol was approved by the Institutional Review Board (IRB) (No. H-2104-126-1213). The requirement for informed patient consent was waived by the IRB as this was a retrospective study, and analyses were performed using de-identified data.

## Results

Baseline characteristics of the patients were compared by weight change: weight loss, stable weight, and weight gain (**Table 1**). There were no significant differences in age, sex, preoperative weight and BMI among the three groups. The prevalence of DM was highest in the weight gain group among the three groups (42.3%, 17.2%, and 3.4%, respectively, P=0.002). In contrast, the prevalence of other comorbidities, such as hypertension, dyslipidemia, cardiovascular disease, stroke, osteoporosis, sleep apnea, arthralgia, and carpal tunnel syndrome, did not differ among the three groups. There were no significant differences in tumor characteristics, including tumor size, GH, and IGF-1 levels (× ULN), among the three groups. However, the absolute level of IGF-1 (ng/mL) of the weight loss group was significantly higher than that of the stable group (843.5 ng/mL [637.5–1095.0 ng/mL] vs. 720.0 ng/mL [505.0–856.0 ng/mL], P=0.039). The remission rates and pre-and postoperative hormone status were similar among the three groups, and body weight change was not influenced by whether remission was achieved. More patients with CDI belonged to the weight loss group compared to the weight gain group (27.6% vs. 0.0%, P=0.004).

**Table 1:** Baseline characteristics of acromegaly patients according to the weight change.

	Weight loss (N=29)	Stable (N=58)	Weight gain (N=26)	*P*
Weight change (kg)	-3.8 [-5.4–2.4]	-0.4 [-1.1–0.8]	3.4 [2.3–5.5]	<0.001^abc^
Percent weight change (%)	-4.6 [-7.2–4.0]	-0.5 [-1.7–1.2]	5.0 [3.7–7.7]	<0.001^abc^
Age at diagnosis (years)	34.0 [28.0–51.0]	46.5 [34.0–56.0]	44.0 [34.0–54.0]	0.065
Male (n,%)	11 (37.9%)	23 (39.7%)	16 (61.5%)	0.128
Height (cm)	166.8 [161.1–178.4]	165.2 [160.0–173.5]	170.6 [161.5–177.7]	0.423
Weight (kg)	74.4 [62.5–82.8]	69.7 [62.6–76.3]	70.6 [62.0–82.2]	0.851
BMI (kg/m^2^)	25.3 [23.5–26.8]	24.1 [23.1–26.9]	23.9 [22.5–27.8]	0.597
SBP (mmHg)	126.0 ± 12.4	123.8 ± 10.6	124.0 ± 8.1	0.468
DBP (mmHg)	77.6 ± 11.1	79.1 ± 9.2	78.5 ± 8.7	0.718
***Comorbidities***				
Diabetes mellitus (n,%)	1 (3.4%)	10 (17.2%)	11 (42.3%)	0.002
HTN (n,%)	8 (27.6%)	18 (31.0%)	12 (46.2%)	0.290
Dyslipidemia (n,%)	2 (6.9%)	6 (10.3%)	6 (23.1%)	0.160
CVD (n,%)	0 (0.0%)	3 (5.2%)	1 (3.8%)	0.467
Stroke (n,%)	1 (3.4%)	0 (0.0%)	0 (0.0%)	0.232
Osteoporosis (n,%)	2 (6.9%)	9 (15.5%)	4 (15.4%)	0.502
Sleep apnea (n,%)	3 (10.3%)	7 (12.1%)	6 (23.1%)	0.324
Arthralgia (n,%)	3 (10.3%)	4 (6.9%)	4 (15.4%)	0.475
Carpal tunnel syndrome (n,%)	3 (10.3%)	3 (5.2%)	1 (3.8%)	0.546
***Tumor characteristics***				
Maximal tumor size (cm)	1.8 [1.3–2.6]	1.7 [1.2–2.6]	1.8 [1.4–2.7]	0.838
Baseline GH (ng/ml)	21.4 [16.2–47.9]	12.2 [7.4–29.7]	18.8 [7.8–24.2]	0.069
Nadir GH during OGTT (ng/ml)	16.5 [7.1–28.2]	8.5 [4.6–21.2]	13.5 [6.4–20.3]	0.299
IGF1 (ng/ml)	843.5 [637.5–1095.0]	720.0 [505.0–856.0]	757.5 [539.0–1230.0]	0.039^a^
IGF1(x ULN)	2.4 [1.8–3.3]	2.3 [1.8–2.9]	2.7 [2.0–3.6]	0.118
***Preoperative hormone status***				
TSH deficiency (n,%)	2 (6.9%)	6 (10.3%)	2 (7.7%)	0.843
ACTH deficiency (n,%)	0 (0.0%)	5 (8.6%)	0 (0.0%)	0.084
FSH/LH deficiency (n,%)	11 (37.9%)	22 (37.9%)	11 (42.3%)	0.923
***Postoperative hormone status***				
Remission (n,%)	21 (72.4%)	46 (79.3%)	20 (76.9%)	0.771
Baseline GH (ng/ml)	1.0 [0.2–3.5]	0.6 [0.2–1.4]	0.7 [0.2–2.8]	0.332
Nadir GH during OGTT (ng/ml)	0.2 [0.2–0.7]	0.1 [0.1–0.5]	0.2 [0.1–0.7]	0.481
IGF1 (ng/ml)	269.5 [205.0–310.0]	231.0 [165.0–319.0]	366.0 [168.0–526.0]	0.201
IGF1(x ULN)	0.7 [0.5; 1.1]	0.7 [0.6; 1.0]	1.0 [0.7; 1.5]	0.133
TSH deficiency (n,%)	3 (10.3%)	5 (8.6%)	2 (7.7%)	0.938
ACTH deficiency (n,%)	2 (6.9%)	3 (5.2%)	2 (7.7%)	0.892
FSH/LH deficiency (n,%)	10 (34.5%)	15 (25.9%)	9 (34.6%)	0.603
Central diabetes insipidus (n,%)	8 (27.6%)	5 (8.6%)	0 (0.0%)	0.004
***Metabolic and biochemical parameters***				
HbA1c (%)	5.9 [5.7–6.1]	5.8 [5.6–6.3]	6.0 [5.6–7.0]	0.155
Fasting plasma glucose (mg/dL)	104.5 [94.0–111.0]	103.0 [92.0–112.5]	110.0 [96.0–123.0]	0.144
1hr-postprandial plasma glucose (mg/dL)	195.0 [143.0–228.5]	181.0 [147.5–217.5]	230.0 [176.0–278.0]	0.020^bc^
2hr-postprandial plasma glucose (mg/dL)	129.0 [112.0–157.0]	138.5 [116.0–158.5]	195.5 [133.0–262.0]	0.016^bc^
Insulin (mlU/ml)*	16.5 [8.1–21.1]	14.5 [10.5–22.8]	14.1 [9.9–17.9]	0.734
HOMA-IR*	3.6 [2.0–4.8]	3.6 [2.6–5.3]	4.3 [2.7–5.3]	0.720
Total cholesterol (mg/dL)	180.5 [167.0–198.5]	173.0 [152.0–198.0]	170.0 [154.0–195.0]	0.522
Serum creatinine (mg/dL)	0.6 [0.5–0.8]	0.6 [0.5–0.7]	0.7 [0.6–0.8]	0.158
Glomerular filtration rate (mL/min/1.73m^2^)	116.1 [108.8–135.5]	117.3 [103.6–135.6]	113.6 [97.3–130.9]	0.611

Variables for categorical variables are presented as n (%); for continuous variables, as mean ± standard deviation or median (IQR). Variables of each group were compared by one-way analysis of variance and the chi-square test.

a P < 0.05 between weight loss group and stable group.

b P < 0.05 between weight loss group and weight gain group.

c P < 0.05 between stable group and weight gain group.

*Data are not available in 52 patients for insulin.

ACTH, adrenocorticotropic hormone; BMI, body mass index; CVD, cardiovascular disease; DBP, diastolic blood pressure; FSH, follicle-stimulating hormone; GH, growth hormone; HbA1c, glycated hemoglobin; HDL, high-density lipoprotein; HOMA-IR, homeostatic model assessment-insulin resistance; HTN, hypertension; IGF 1, insulin like growth factor 1; LDL, low-density lipoprotein; LH, luteinizing hormone; OGTT, oral glucose tolerance test; SBP, systolic blood pressure; TSH, thyroid stimulating hormone; ULN, upper limit of normal.

When the metabolic and biochemical parameters of the three groups were compared, HbA1c, fasting plasma glucose, and insulin resistance (HOMA-IR) were not significantly different between the three groups. However, 1 h- and 2 h-postprandial plasma glucose levels were significantly higher in the weight gain group than in the other two groups. Baseline body composition did not differ among the three groups (**Supplemental Table 1**).

**Figure 1:**
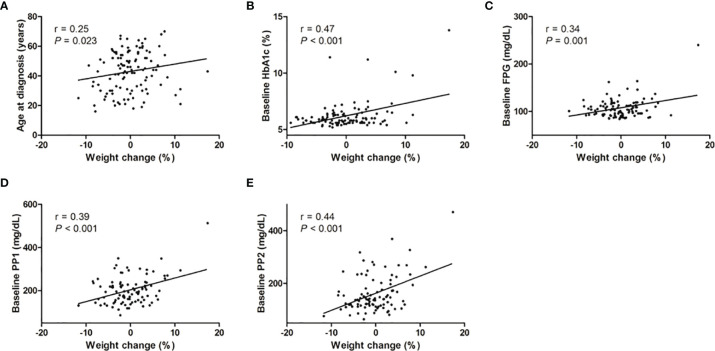
Correlation analysis between percent weight change and other parameters. The correlation between percent weight change and clinical parameters was analyzed by Pearson and Spearman correlation analysis. **(A)** Age at diagnosis, **(B)** baseline HbA1c, **(C)** FPG, **(D)** PP1, and **(E)** PP2 were significantly positively correlated with percentage weight change in acromegalic patients. FPG, fasting plasma glucose; PP1, 1hr-postprandial plasma glucose; PP2, 2hr-postprandial plasma glucose.

We further conducted a correlation analysis between the percent weight change and clinical parameters (**Figure 1** and **Supplemental Table 2**). Postoperative weight change was positively correlated with the age at diagnosis (r=0.25, P=0.023). However, there was no correlation between preoperative weight, BMI, GH levels, IGF-1 levels, and weight gain. In contrast, HbA1c (r=0.47, P<0.001), fasting plasma glucose (r=0.34, P=0.001), 1 h-postprandial plasma glucose (r=0.39, P<0.001), and 2 h-postprandial plasma glucose (r=0.44, P<0.001) were significantly correlated with postoperative weight change. However, HOMA-IR was not related to postoperative weight changes.

**Figure 2:**
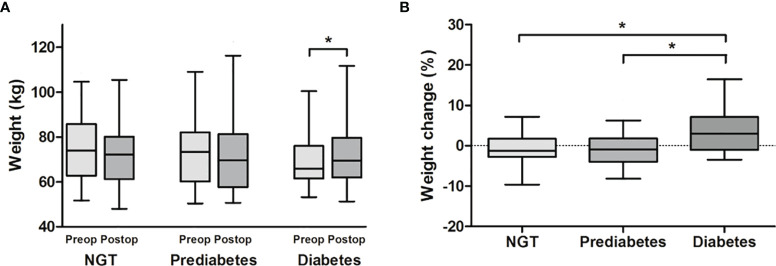
Comparison of **(A)** weight and **(B)** weight change according to the glycemic status at baseline. Acromegaly patients were divided into NGT, prediabetes, and diabetes groups. **(A)** Body weight before and after surgery was compared by paired t-test, and **(B)** postoperative weight change (%) were compared by one-way analysis of variance. There was a significant weight gain after surgery only in the diabetes group. Postoperative weight gain (%) was significantly greater in the diabetes group than in the NGT and prediabetes groups, respectively. *P < 0.05 between each group. NGT, normal glucose tolerance.

Since the presence of DM at baseline was the only significantly different variable among the three acromegaly groups according to weight change, we further investigated the weight and body composition parameters according to the glycemic status at baseline. The prevalence of prediabetes and DM was 47.8 and 19.5%, respectively. Weight gain after surgery was significantly higher in the DM group than the prediabetes and NGT groups (3.0% [-1.0%–7.2%], -0.9% [-4.0%–1.8%], and -1.2% [-2.7–1.7%], respectively), although there was no significant difference in preoperative body weight among the three groups (**Figure 2**). We further performed logistic regression analysis for postoperative weight gain according to the glycemic status (**Table 2**). The risk for postoperative weight gain was significantly higher in the DM group than in the NGT group after adjustment for age, IGF-1, and postoperative CDI (OR 7.65 [1.81–32.4]). Moreover, a 1% increase in HbA1c elevated the risk of postoperative weight gain by 1.6-fold.

**Table 2:** Odds ratios of the prediabetes and diabetes group for risk of postoperative weight gain compared with the NGT group.

Variables	Univariate model		Multivariate model	
	OR [95% CI]	P	OR [95% CI]	P
Glycemic status				
NGT	1.00	–	1.00	–
Prediabetes	1.03 [0.33–3.20]	0.955	1.18 [0.36–3.88]	0.787
Diabetes mellitus	5.17 [1.54–17.32]	0.008	7.65 [1.81–32.39]	0.006
HbA1c (per 1%)	1.70 [1.12–2.59]	0.013	1.64 [1.08–2.50]	0.021

Logistic regression analysis was performed to analyze the degree to which glycemic status predicts postoperative weight gain. Results were expressed as odds ratios and 95% confidence intervals. In the univariate model, both prediabetes and diabetes significantly predicted postoperative weight gain, and the significance was maintained even after adjusting for age, IGF1 (xULN) and postoperative CDI status. And for each 1% HbA1c increment, the risk of postoperative weight gain increased by 1.7 times.

CDI, central diabetes mellitus; CI, confidence interval; IGF1, insulin-like growth factor 1; NGT, normal glucose tolerance; OR, odds ratio; ULN, upper limit of normal.

As shown in **Table 3**, baseline GH, nadir GH during OGTT, and IGF-1 in all three groups decreased significantly after surgery. In the prediabetes and DM groups, HbA1c and fasting plasma glucose levels significantly improved after surgery. When comparing the postoperative changes in body composition among the three groups (**Table 4**), waist circumference significantly increased only in the DM group; however, the waist-to-hip ratio increased in the prediabetes and DM groups. The lean mass and percentage lean mass decreased in the prediabetes and DM groups, while the fat mass, percentage fat mass, and visceral fat area significantly increased after surgery. However, the NGT group did not show any changes in body composition pre- and post-surgery.

**Table 3:** Change of metabolic parameters and body composition in patients with acromegaly according to the glycemic status at baseline.

	NGT (N=37)			Prediabetes (N=54)			Diabetes (N=22)		
	Preop	Postop	*P*	Preop	Postop	*P*	Preop	Postop	*P*
Baseline GH (ng/ml)	18.7 [10.8–30.4]	0.6 [0.2–1.1]	<0.001	17.6 [7.0–38.2]	1.2 [0.2–2.9]	<0.001	16.6 [8.0–32.6]	0.6 [0.2–1.9]	<0.001
Nadir GH during OGTT (ng/ml)	10.1 [4.7–24.7]	0.2 [0.1–0.5]	<0.001	10.6 [5.6–20.3]	0.3 [0.1–0.8]	<0.001	13.5 [6.6–19.4]	0.2 [0.1–0.7]	<0.001
IGF1 (ng/ml)	469.0 [579.0–1090.0]	276.0 [204.0–385.0]	<0.001	757.5 [545.0–943.5]	240.5 [157.0–377.8]	<0.001	629.0 [523.5–896.0]	232.0 [151.5–376.5]	<0.001
IGF1 (x ULN)	2.4 [1.8–3.0]	0.8 [0.6–1.1]	<0.001	2.3 [1.7–3.2]	0.7 [0.5–1.2]	<0.001	2.4 [2.0–3.0]	0.9 [0.6–1.4]	<0.001
SBP (mmHg)	124.4 ± 9.4	123.6 ± 11.7	0.701	124.9 ± 11.8	119.2 ± 11.3	<0.001	123.4 ± 9.1	123.9 ± 12.7	0.815
DBP (mmHg)	75.6 ± 10.6	76.3 ± 8.9	0.658	79.5 ± 9.2	76.1 ± 9.0	0.005	81.1 ± 7.4	79.6 ± 9.6	0.286
HbA1c (%)	5.4 [5.4–5.6]	5.7 [5.4–5.9]	0.343	6.1 [5.9–6.3]	5.7 [5.4–5.8]	0.001	6.9 [6.6–7.5]	6.2 [5.9–6.4]	<0.001
Fasting plasma glucose (mg/dL)	96.0 [89.0–104.0]	87.0 [81.0–94.5]	0.001	106.5 [96.3–112.0]	91.0 [85.0–100.3]	<0.001	118.0 [109.0–141.5]	100.0 [93.5–111.0]	0.001
Total cholesterol (mg/dL)	182.5 [165.3–198.0]	181.0 [148.8–206.8]	0.894	173.0 [163.5–210.5]	180.0 [150.5–202.0]	0.104	161.0 [143.5–202.0]	183.0 [136.5–208.0]	0.570

Variables are presented as mean ± standard deviation or median (IQR).

Variables were compared by paired t-test and Wilcoxon singed rank test.

DBP, diastolic blood pressure; GH, growth hormone; HbA1c, glycated hemoglobin; HDL, high-density lipoprotein; IGF1, insulin-like growth factor 1; LDL, low-density lipoprotein; OGTT, oral glucose tolerance test; SBP, systolic blood pressure.

**Table 4:** Postoperative change of body composition parameters in patients with acromegaly according to the glycemic status at baseline.

	NGT (N=7)			Prediabetes (N=16)			Diabetes (N=9)		
	Preop	Postop	*P*	Preop	Postop	*P*	Preop	Postop	*P*
Waist circumference (cm)	80.5 ± 9.9	79.0 ± 10.4	0.237	85.3 ± 9.6	88.1 ± 9.1	0.060	86.2 ± 7.2	91.8 ± 7.3	0.002
Hip circumference (cm)	96.5 ± 6.1	94.2 ± 5.8	0.097	99.4 ± 7.9	98.6 ± 8.5	0.456	96.0 ± 3.6	98.4 ± 4.5	0.058
Waist-to-hip ratio	0.86 [0.79–0.88]	0.87 [0.86–0.89]	0.141	0.85 [0.81–0.88]	0.89 [0.84–0.91]	0.007	0.85 [0.81–0.89]	0.89 [0.87–0.95]	0.018
Weight (kg) *	73.6 ± 14.8	72.9 ± 2.4	0.209	72.5 ± 2.1	71.8 ± 2.2	0.156	69.3 ± 2.5	71.7 ± 2.9	0.008
Lean mass (kg)	25.5 [22.2–39.6]	25.0 [22.1–35.1]	0.176	26.8 [22.1–38.4]	24.8 [22.4–35.8]	0.002	33.3 [27.0–37.7]	32.5 [25.7–36.5]	0.008
Percentage lean mass (%)	45.5 [40.4–48.5]	43.3 [40.7–46.3]	0.310	44.2 [36.7–48.0]	42.4 [34.9–44.8]	0.001	46.9 [43.9–49.9]	43.2 [40.5–46.3]	0.008
Fat mass (kg)	12.3 [8.0–16.2]	14.3 [11.7–16.1]	0.237	12.1 [10.0–16.4]	16.4 [13.7–19.8]	0.003	10.5 [8.1–14.3]	17.6 [13.5–19.7]	0.008
Percentage fat mass (%)	16.4 [14.4–25.7]	21.8 [17.2–26.1]	0.237	19.0 [15.5–31.6]	23.4 [20.7–35.2]	<0.001	16.3 [10.9–20.6]	22.0 [17.4–27.6]	0.012
Visceral fat area (cm^2^)	54.9 [32.1–72.5]	65.6 [55.4–67.6]	0.397	52.9 [41.4–65.9]	70.7 [60.4–86.8]	0.008	48.6 [40.0–74.1]	81.0 [60.9–90.3]	0.011

Variables are presented as mean ± standard deviation or median (IQR).

Variables were compared by paired t-test and Wilcoxon signed rank test.

*Data are available in 37 patients in NGT group, 54 in prediabetes group and 22 in diabetes group.

DBP, diastolic blood pressure; GH, growth hormone; HbA1c, glycated hemoglobin; HDL, high-density lipoprotein; IGF1, insulin-like growth factor 1; LDL, low-density lipoprotein; OGTT, oral glucose tolerance test; SBP, systolic blood pressure.

## Discussion

In the present study, 23% of patients with acromegaly experienced a weight gain of ≥ 3% after surgery. The predictors for postoperative weight gain were DM at diagnosis and the absence of CDI. Further, the degree of weight gain after surgery was positively correlated with the baseline glycemic status in patients with acromegaly. Moreover, increased fat mass, i.e., visceral fat mass, contributed to postoperative weight gain in patients with prediabetes and DM despite decreased lean mass.

Previous studies have reported that biochemical control after surgery leads to increased fat mass and decreased muscle mass despite improved glycemic status and insulin resistance in patients with acromegaly (15, 16, 24). This study showed similar changes in body weight and composition postoperatively. The median weight change before and after surgery was 0.5% (-3.2 ~ 2.7%), and percentage fat mass and lean mass were 22.4 (10.9–43.6%) and -4.9% (-6.6% –2.1%), respectively, in all patients.

As an anabolic hormone, GH affects glucose and lipid metabolism, counteracting insulin action. GH increases free fatty acids in the blood by inducing lipolysis and lipid oxidation, which reduces glucose uptake in peripheral tissues (25). Therefore, subcutaneous and visceral fat mass is reduced, and intramuscular fat mass is increased in patients with active acromegaly (19, 26). Elevated free fatty acids also exhibit oxidative stress and proinflammatory effects (27). Chronic inflammation caused by free fatty acids can result in insulin resistance; this occurs by disrupting the insulin signaling pathway by degrading the phosphorylated insulin receptor substate-1 (28–30). Therefore, patients with acromegaly exhibit increased insulin resistance despite a decrease in fat mass and an increase in muscle mass. Surgical intervention for excessive GH, which lowers the elevated resting energy expenditure and lipolysis rates, may explain increases in weight and fat mass (17).

Some studies have identified that GH or IGF-1 levels or hypogonadism are correlated with fat and lean mass. However, this is inconsistent with our study findings (17, 24). The remission rate and postoperative hormone status, including nadir GH and IGF-1 levels and hypogonadism, were not different among the three groups in our study. Hence, other factors may contribute to weight gain after surgery in patients with acromegaly.

The present study shows that DM at diagnosis and glycemic status significantly contributed to changes in body composition after surgery. In addition, age at diagnosis was correlated with postoperative weight gain in our study. In acromegaly, insulin resistance due to the insulin-antagonistic effects of excess GH is compensated for by an increase in β-cell function and insulin secretion (7). However, prolonged disease duration may result in the exhaustion of β-cell secretory function and the development of overt DM in patients with acromegaly. We also observed that insulin levels were lower in patients with DM (**Supplemental Table 3**). Therefore, DM can persist in patients with acromegaly, even after surgical remission, due to the irreversible change in insulin secretion and sensitivity (13, 31). Hence, older patients with DM and acromegaly may exhibit the typical body composition phenotype of metabolic syndrome since ‘metabolic memory’ remains in patients with DM (32). Likewise, insulin resistance may persist even after normalizing GH in acromegalic patients with DM. In addition, hyperinsulinemia induced by insulin resistance may increase fat due to the anabolic effect of insulin (33).

Furthermore, an imbalance in adipokines in patients with DM may increase adiposity (34, 35). Ghrelin and leptin levels decrease, while adiponectin levels increase in patients with acromegaly, reflecting a decrease in healthy adipose tissue (15, 25, 36). After surgery, ghrelin levels increase due to GH normalization and improved insulin sensitivity, but leptin levels also increase, which is associated with the development of insulin resistance (15, 37). Increased adiponectin levels after surgery may be associated with an insulin-sensitizing effect in patients with acromegaly (38). Therefore, the imbalance of adipokines may be further altered in patients with DM and acromegaly, who may present with more significant weight gain after surgery (36).

On the other hand, it is controversial whether fat mass gain after disease control directly contributes to increased cardiovascular risk. Some reports have revealed no cardiovascular events after the treatment of acromegaly (21). However, other studies have noted that complications such as hypertension, DM, and obstructive sleep apnea did not improve after treating acromegaly (13, 14). Increased visceral and subcutaneous fat can offset the effect of improvement of metabolic complications caused by decreased GH in patients with acromegaly. Moreover, subcutaneous fat accumulation may aggravate facial and musculoskeletal disfigurement, which impairs quality of life and body image perception in patients with acromegaly (39).

This study has several limitations. As a retrospective study, there were missing values of evaluation items such as insulin and body composition. The number of study subjects was small, and the follow-up period was short. We used bioimpedance analysis rather than dual-energy X-ray absorptiometry to assess body composition. Bioimpedance analysis assumes a constant hydration status, but excess GH results in water retention and edema. Thus, skeletal muscle mass assessment may not be accurate, but fat mass assessment may be acceptable as it is not affected by water content (40). In addition, HbA1c and fasting plasma glucose levels were not significantly different among the three groups, although they were significantly related to the postoperative weight change. This might be attributed to the small number of subjects per group, *i.e.*, the weight gain. Although it was not statistically significant, HbA1c and fasting plasma glucose levels were higher in the weight gain group. Antidiabetic medications can affect postoperative weight change. However, due to the low prevalence of DM and the use of antidiabetic medications in our study subjects (n=14), the effect of antidiabetic medications on body composition or insulin resistance could not be analyzed. Finally, although we assumed that the development of diabetes in acromegalic patients might be attributed to disease duration and individual responsiveness to GH as well as GH excess, we failed to explain the exact mechanism for more postoperative weight gain in patients with acromegaly and diabetes.

However, a strength of the present study is the identification of the high-risk group for weight gain after surgery in patients with acromegaly. Patients with acromegaly and DM have a greater postoperative weight gain and are prone to increasing fat mass, i.e., visceral fat. Although DM improved after disease control of acromegaly, there is a residual cardiovascular risk and impaired quality of life due to weight and fat gain after surgery. Accordingly, preoperative intensive glycemic control and postoperative weight control should be emphasized for patients with acromegaly and DM for better long-term prognosis.

## Data Availability Statement

The original contributions presented in the study are included in the article/**Supplementary Material**. Further inquiries can be directed to the corresponding author.

## Ethics Statement

The studies involving human participants were reviewed and approved by Institutional Review Board of Seoul National University Hospital. Written informed consent for participation was not required for this study in accordance with the national legislation and the institutional requirements.

## Author Contributions

Conception or design: JHK. Acquisition, analysis, or interpretation of data: HNJ and JHK. Drafting the work or revising: HNJ, YHK, and JHK. All authors contributed to the article and approved the submitted version.

## Funding

This study was supported by the grant (No.: 2017R1D1A1B03031879 to JHK) from National Research Foundation by the Ministry of Science, ICT and Future Planning, Republic of Korea.

## Conflict of Interest

The authors declare that the research was conducted in the absence of any commercial or financial relationships that could be construed as a potential conflict of interest.

## Publisher’s Note

All claims expressed in this article are solely those of the authors and do not necessarily represent those of their affiliated organizations, or those of the publisher, the editors and the reviewers. Any product that may be evaluated in this article, or claim that may be made by its manufacturer, is not guaranteed or endorsed by the publisher.
