# Supplementary material for: Diabetes Mellitus Predicts Weight Gain After Surgery in Patients With Acromegaly
Source: Front Endocrinol (Lausanne). 2022 Mar 9;13:854931. doi: 10.3389/fendo.2022.854931 (PMC8959539; doi:10.3389/fendo.2022.854931)
Supplement: Supplementary file 1 [file Table_1.docx]

Supplementary Material

Supplemental Table 1. Body composition of acromegaly patients according to the weight change

|  | Weight loss  (N=19) | Stable  (N=33) | Weight gain  (N=19) | *P* |
| --- | --- | --- | --- | --- |
| Waist circumference (cm) | 84.4 ± 5.8 | 84.2 ± 8.0 | 84.7 ± 10.3 | 0.905 |
| Hip circumference (cm) | 100.3 ± 6.5 | 98.7 ± 6.6 | 96.4 ± 5.5 | 0.064 |
| Waist-to-hip ratio | 0.8 [0.8–0.9] | 0.8 [0.8–0.9] | 0.9 [0.8–0.9] | 0.395 |
| Lean mass (kg) | 36.0 [26.8–41.0] | 29.4 [23.7–36.2] | 30.5 [26.2–35.8] | 0.254 |
| Percentage lean mass (%) | 47.8 [41.9–50.0] | 44.8 [40.4–48.5] | 45.5 [42.8–46.9] | 0.535 |
| Fat mass (kg) | 11.8 [9.8–14.5] | 13.4 [10.1–16.2] | 11.6 [9.6–14.1] | 0.618 |
| Percentage fat mass (%) | 15.6 [11.2–23.4] | 19.2 [14.2–25.7] | 18.1 [15.4–23.3] | 0.581 |
| Visceral fat area (cm^2^) | 54.9 [46.5–67.2] | 64.4 [45.7–72.5] | 50.3 [42.0–67.2] | 0.763 |

Variables for categorical variables are presented as mean ± standard deviation or median (IQR).

Variables of each group were compared by one-way analysis of variance.

Supplemental Table 2. Correlation analysis between percent weight change and other parameters

| Variables | *r* | *P* |
| --- | --- | --- |
| **Age at diagnosis (years)** | **0.25** | **0.023** |
| Baseline weight (kg) | 0.00 | 0.978 |
| Baseline BMI (kg/m^2^) | -0.06 | 0.583 |
| Baseline nadir GH (ng/mL) | -0.04 | 0.737 |
| Baseline IGF-1 (ng/mL) | 0.13 | 0.231 |
| IGF-1 change (%) | 0.10 | 0.347 |
| Nadir GH change (%) | 0.06 | 0.597 |
| **Baseline HbA1c (%)** | **0.47** | **<0.001** |
| **Baseline fasting plasma glucose (mg/dL)** | **0.34** | **0.001** |
| **Baseline 1hr-postprandial plasma glucose (mg/dL)** | **0.39** | **<0.001** |
| **Baseline 2hr-postprandial plasma glucose (mg/dL)** | **0.44** | **<0.001** |
| Baseline HOMA-IR | 0.05 | 0.698 |

Correlation analysis was performed by Pearson and Spearman analysis.

BMI, body mass index; GH, growth hormone; HbA1c, glycated hemoglobin; HOMA-IR, homeostatic model assessment-insulin resistance; IGF-1, insulin-like growth factor 1.

Supplemental Table 3. Clinical and biochemical characteristics of acromegaly patients according to the glycemic status

|  | NGT (N=37) | Prediabetes (N=54) | Diabetes (N=22) | *P* |
| --- | --- | --- | --- | --- |
| Age at diagnosis (years) | 38.0 [28.0–46.0] | 44.5 [30.0–55.0] | 53.0 [48.0–60.0] | **<0.001** |
| Male (n,%) | 22 (59.5%) | 17 (31.5%) | 11 (50.0%) | **0.026** |
| Height (cm) | 172.1  [164.4–178.1] | 164.2  [158.7–171.9] | 168.0  [160.9–172.0] | **0.042** |
| Weight (kg) | 73.9 [62.8–85.4] | 73.3 [61.1–82.1] | 66.0 [62.0–75.4] | 0.614 |
| BMI (kg/m^2^) | 24.2 [22.4–26.8] | 25.1 [23.2–28.0] | 23.5 [22.7–25.0] | 0.208 |
| SBP (mmHg) | 124.4 ± 9.4 | 124.8 ± 11.9 | 123.3 ± 8.9 | 0.761 |
| DBP (mmHg) | 75.6 ± 10.6 | 79.7 ± 9.2 | 80.8 ± 7.4 | **0.026** |
| ***Comorbidities*** |  |  |  |  |
| HTN (n,%) | 9 (24.3%) | 15 (27.8%) | 14 (63.6%) | **0.004** |
| Dyslipidemia (n,%) | 1 (2.7%) | 7 (13.0%) | 6 (27.3%) | **0.048** |
| CVD (n,%) | 0 (0.0%) | 2 (3.7%) | 2 (9.1%) | 0.188 |
| Stroke (n,%) | 0 (0.0%) | 0 (0.0%) | 1 (4.5%) | 0.124 |
| Osteoporosis (n,%) | 2 (5.4%) | 10 (18.5%) | 3 (13.6%) | 0.194 |
| Sleep apnea (n,%) | 5 (13.5%) | 7 (13.0%) | 4 (18.2%) | 0.831 |
| Arthralgia (n,%) | 2 (5.4%) | 6 (11.1%) | 3 (13.6%) | 0.526 |
| Carpal tunnel syndrome (n,%) | 2 (5.4%) | 5 (9.3%) | 0 (0.0%) | 0.306 |
| ***Tumor characteristics*** |  |  |  |  |
| Maximal tumor size (cm) | 1.8 [1.4–2.8] | 1.7 [1.3–2.6] | 1.6 [1.1–2.2] | 0.309 |
| Baseline GH (ng/ml) | 18.7 [11.1–30.4] | 17.6 [7.0–36.7] | 16.6 [8.1–32.4] | 0.803 |
| Nadir GH during OGTT (ng/ml) | 9.6 [5.3–24.1] | 11.3 [4.6–20.9] | 12.5 [6.6–19.4] | 0.876 |
| IGF1 (ng/ml) | 808.0  [598.0–1090.0] | 758.0  [569.0–952.0] | 629.0  [525.0–804.0] | 0.371 |
| IGF1(x ULN) | 2.5 [1.8–3.0] | 2.3 [1.8–3.2] | 2.4 [2.0–3.0] | 0.985 |
| ***Preoperative hormone status*** |  |  |  |  |
| TSH deficiency (n,%) | 1 (2.7%) | 6 (11.1%) | 3 (13.6%) | 0.259 |
| ACTH deficiency (n,%) | 2 (5.4%) | 1 (1.9%) | 2 (9.1%) | 0.357 |
| FSH/LH deficiency (n,%) | 14 (37.8%) | 22 (40.7%) | 8 (36.4%) | 0.926 |
| ***Postoperative hormone status*** | | | | |
| Remission (n,%) | 12 (32.4%) | 24 (44.4%) | 9 (40.9%) | 0.513 |
| Baseline GH (ng/ml) | 0.6 [0.2–0.9] | 1.2 [0.2–2.8] | 0.6 [0.2–1.5] | 0.217 |
| Nadir GH during OGTT (ng/ml) | 0.1 [0.1–0.5] | 0.3 [0.1–0.7] | 0.2 [0.1–0.5] | 0.455 |
| IGF1 (ng/ml) | 276.0  [204.5–368.5] | 245.0  [157.0–338.0] | 240.5  [157.0–367.0] | 0.614 |
| IGF1(x ULN) | 0.8 [0.6–1.1] | 0.7 [0.5–1.1] | 0.9 [0.6–1.4] | 0.623 |
| TSH deficiency (n,%) | 2 (5.4%) | 6 (11.1%) | 2 (9.1%) | 0.641 |
| ACTH deficiency (n,%) | 2 (5.4%) | 3 (5.6%) | 2 (9.1%) | 0.821 |
| FSH/LH deficiency (n,%) | 8 (21.6%) | 20 (37.0%) | 6 (27.3%) | 0.275 |
| Central diabetes insipidus (n,%) | 4 (10.8%) | 8 (14.8%) | 1 (4.5%) | 0.439 |
| ***Metabolic and biochemical parameters*** | | | | |
| HbA1c (%) | 5.6 [5.4–5.6] | 5.9 [5.8–6.1] | 7.0 [6.6–7.5] | < 0.001 |
| Fasting plasma glucose (mg/dL) | 93.0  [91.0–102.0] | 105.0  [96.0–112.0] | 118.0  [110.0–137.0] | < 0.001 |
| 1hr-postprandial plasma glucose (mg/dL) | 157.0  [137.0–176.0] | 196.0  [160.0–223.5] | 274.0  [230.0–303.0] | < 0.001 |
| 2hr-postprandial plasma glucose (mg/dL) | 116.0  [103.5–138.5] | 140.0  [120.5–167.5] | 241.0  [185.0–284.0] | < 0.001 |
| Insulin (mlU/ml) | 17.1 [12.2–20.9] | 17.9 [11.1–26.4] | 9.8 [8.5–13.1] | **0.003** |
| HOMA-IR | 4.0 [2.8–5.1] | 4.5 [2.8–7.0] | 2.7 [2.5–3.1] | 0.054 |
| Total cholesterol (mg/dL) | 180.5  [162.0–197.5] | 177.0  [165.0–204.0] | 161.0  [144.0–188.0] | 0.142 |
| Serum creatinine (mg/dL) | 0.7 [0.6–0.8] | 0.6 [0.5–0.7] | 0.6 [0.5–0.8] | 0.098 |
| Glomerular filtration rate (mL/min/1.73m^2^) | 114.7  [103.5–130.6] | 116.5  [106.3–141.4] | 117.0  [99.1–125.6] | 0.671 |

Variables for categorical variables are presented as n (%); for continuous variables, as mean ± standard deviation or median (IQR). Variables of each group were compared by one-way analysis of variance and the chi-square test.

ACTH, adrenocorticotropic hormone; BMI, body mass index; CVD, cardiovascular disease; DBP, diastolic blood pressure; FSH, follicle-stimulating hormone; GH, growth hormone; HbA1c, glycated hemoglobin; HDL, high-density lipoprotein; HOMA-IR, homeostatic model assessment-insulin resistance; HTN, hypertension; IGF 1, insulin-like growth factor 1; LDL, low-density lipoprotein; LH, luteinizing hormone; OGTT, oral glucose tolerance test; SBP, systolic blood pressure; TSH, thyroid-stimulating hormone; ULN, upper limit of normal.
